# Density patterns and age structure of the queen conch (*Aliger gigas*) in a coral reef lagoon in the Mexican Caribbean

**DOI:** 10.7717/peerj.21836

**Published:** 2026-10-27

**Authors:** Isvi Areli Benítez-Castillo, José Manuel Castro-Pérez, Alejandro Medina Quej, Sergio Cohuo-Duran, Gilberto Acosta-González

**Affiliations:** 1 Tecnológico Nacional de México, Instituto Tecnológico de Chetumal, Chetumal, QUINTANA ROO, Mexico; 2 Unidad de Ciencias del Agua, CONAHCYT. Water Sciences Unit/Scientific Research Center of Yucatan AC (CICY), Cancun, QUINTANA ROO, Mexico

**Keywords:** Population density, Queen conch, Size and age structure, Banco Chinchorro, México

## Abstract

The queen conch (*Aliger gigas*) is one of the most important fishery resources in the Caribbean; however, its populations have declined markedly as a result of overexploitation. This study evaluated the population density and size structure of the species within the Banco Chinchorro Biosphere Reserve, Mexico, during 2015, 2016, and 2018, encompassing the final phase of a 5-year total fishing closure and the period immediately following its termination. A total of 35 sampling stations were distributed across six zones of the reef lagoon. At each station, five circular surveys with an 8 m radius were conducted during each sampling campaign. The highest densities were observed in the Central and Southern Leeward zones, whereas the lowest occurred in the Southern Windward zone. Population density increased during the fishing closure and reached its maximum in 2016, suggesting a partial recovery associated with reduced fishing pressure. Despite this increase, the population was dominated by juveniles and subadults, while sexually mature individuals represented only a small proportion of the stock. Spatial variation in size distribution revealed distinct areas functioning as recruitment, growth, and potentially reproductive habitats within the reserve. Conversely, the scarcity of large individuals suggests the long-term effects of selective harvesting on the adult component of the population. Comparison between siphonal length and lip thickness indicated that only 6.2% of individuals simultaneously met the legal minimum harvest size and a morphological criterion associated with sexual maturity, whereas 10.03% attained the minimum legal size without having reached reproductive maturity. These findings indicate that siphonal length alone may not adequately protect the reproductive stock of *A. gigas*. Accordingly, size-based management regulations should be complemented by biological indicators of maturity, such as lip thickness, to enhance conservation measures and promote the sustainable management of queen conch populations in Banco Chinchorro. Collectively, these findings provide a robust scientific baseline for future conservation and management actions aimed at safeguarding reproductive stocks and ensuring the long-term sustainability of *A. gigas* fisheries.

## Introduction

The queen conch (*Aliger gigas*) (Maxwell et al., 2020) is distributed throughout the Caribbean Sea, from southeastern Florida to northern South America, including the Bahamas and Bermuda (Randall, 1964). This herbivorous marine snail occupies a broad range of benthic habitats throughout the Caribbean, including seagrass meadows, sandy substrates, and coral rubble, as well as areas dominated by macroalgae. It is typically found at depths between 2 and 60 m (Ray & Stoner, 1994; Glazer & Kidney, 2004; Horn et al., 2022).

Queen conch fishing has been a key cultural and food practice in the Caribbean since pre-Columbian times and is used in rituals, trade, ornamentation and gastronomy (Marco, Valderrama & Rueda, 2021). Together with the spiny lobster (*Panulirus argus*), it is one of the most important fisheries in the region, with exports exceeding 74 million USD in 2017 (Prada et al., 2017). However, overexploitation has led to a significant decline in their populations, especially in countries where SCUBA diving is used for catching (Vaz et al., 2022; Kough et al., 2025). Consequently, the queen conch was included in Appendix II of CITES in 1992 and was first listed in the IUCN Red List of Threatened Species in 1994 (Groombridge, 1993; Food and Agriculture Organization of the United Nations (FAO), 2001). Since then, its conservation status has been periodically reviewed and reassessed in response to ongoing concerns regarding population declines caused primarily by commercial exploitation. As a response to the decline of queen conch, most Caribbean countries have adopted management measures for its recovery. Out of 30 countries, 15 apply closed seasons from 2 to 6 months, and several such as Cuba, Mexico, Belize and Venezuela have imposed total closures. In addition, 20 countries set minimum shell sizes (17.5–22.9 cm) and 12 require minimum lip thickness (5–10 mm) (Food and Agriculture Organization of the United Nations (FAO), 2001). Fishing with SCUBA equipment is prohibited in 10 countries, and some have set depth limits, catch quotas as well as export restrictions (Appeldoorn & Baker, 2013; Prada et al., 2017).

In Quintana Roo, the exploitation of the queen conch for subsistence purposes began in the 1950s. However, in the 1970s harvest significantly increased (Basurto-Origel et al., 2005). Consequently, during the 1980s and 1990s, natural populations within traditional fishing grounds declined markedly, leading to restrictions that limited harvesting to Cozumel Island and Banco Chinchorro (Basurto-Origel et al., 2000). The latter was designated as the Banco Chinchorro Biosphere Reserve (BCBR) under Mexican law in 1996 (Jordán & Martin, 1987; Basurto-Origel et al., 2000). Since 2006, BCBR has remained the only area in Quintana Roo where legal fishing of queen conch is permitted (Basurto-Origel et al., 2000). Fishing in this place is restricted to free diving, which takes place at depths between 8 and 20 m. On February 13^th^, 2009, temporary closures were implemented (in February and from May to November), while the Mexican Official Standard NOM-013-PESC-1994, a legally binding federal regulation, established minimum sizes, fishing methods and annual quotas, which were reduced from 45 to 12 tonnes in 2011 (García-Rivas et al., 2012). In 2012, a total 5-year moratorium on harvest was decreed for the population to recover. Ever since, legal quotas have ranged from 9 to 10.5 tonnes a year. However, illegal fishing continues unchecked and illegal catches of up to 30 tonnes a year are estimated, exceeding the quotas allowed for cooperatives (García-Rivas et al., 2012).

The queen conch is one of the most extensively studied marine invertebrates in the Mexican Caribbean, including within BCBR. Over the past decades, research has progressively characterized key aspects of its biology and ecology, including spatial distribution and abundance patterns (Domínguez-Viveros, Sosa-Cordero & Medina-Quej, 1994; De Jesús-Navarrete, Medina-Quej & Oliva-Rivera, 2003), growth and mortality processes (Medina-Quej, Domínguez-Viveros & De Jesús-Navarrete, 2005), larval dispersal and recruitment dynamics (Paris et al., 2008; Chávez-Villegas et al., 2012), the genetic structure of regional populations (Zamora-Bustillos et al., 2011; Machkour-M’Rabet et al., 2017), and reproductive patterns (Aldana-Aranda et al., 2003; Cala et al., 2013). In addition, the species has also been used as a bioindicator of environmental contamination, with microplastics detected in queen conch feces using non-invasive sampling techniques (Aldana Aranda et al., 2018, 2022). In parallel, several studies have examined fishery performance and management challenges associated with its exploitation (Basurto-Origel et al., 2010; García-Rivas et al., 2012). Despite the breadth of this literature, however, spatially comprehensive assessments of population density and structure across all suitable habitats within BCBR remain limited. To address this gap, the present study provides an integrated, reef-scale analysis of queen conch population density and age structure within the reserve.

## Materials and Methods

### Area of study

BCBR makes part of the Mesoamerican Reef System, which extends approximately 1,000 km along the coasts of Honduras, Guatemala, Belize, and Mexico. The reef was designated as a marine protected area under the category of Biosphere Reserve in 1996 (Comisión Nacional de Áreas Naturales Protegidas (CONANP), 1996). The reserve is located in southeastern Mexico, in the state of Quintana Roo, 42 km off the coast of Mahahual (18°47′–18°23′ N; 87°14′–87°27′ W) (Comisión Nacional de Áreas Naturales Protegidas (CONANP), 1996) (Fig. 1). The morphology of the atoll and its reef structure have been extensively documented by Chávez & Hidalgo (1984) and Jordán & Martin (1987), who describe its geomorphological composition and note that the system has an oval shape, covering an approximate area of 814.2 km^2^ (45 km in length and 16 km in width). BCBR is composed of three main cays: Cayo Norte (0.4 km^2^), Cayo Centro (5.4 km^2^), and Cayo Lobos (0.1 km^2^). Additionally, González et al. (2003) describe the bathymetry of the reef lagoon, reporting depths ranging from 2 to 12 m and identifying differences in structural complexity between the northern and southern portions of the reef. Together, these physical and environmental characteristics help explain the spatial heterogeneity observed within the study area.

**Figure 1:**
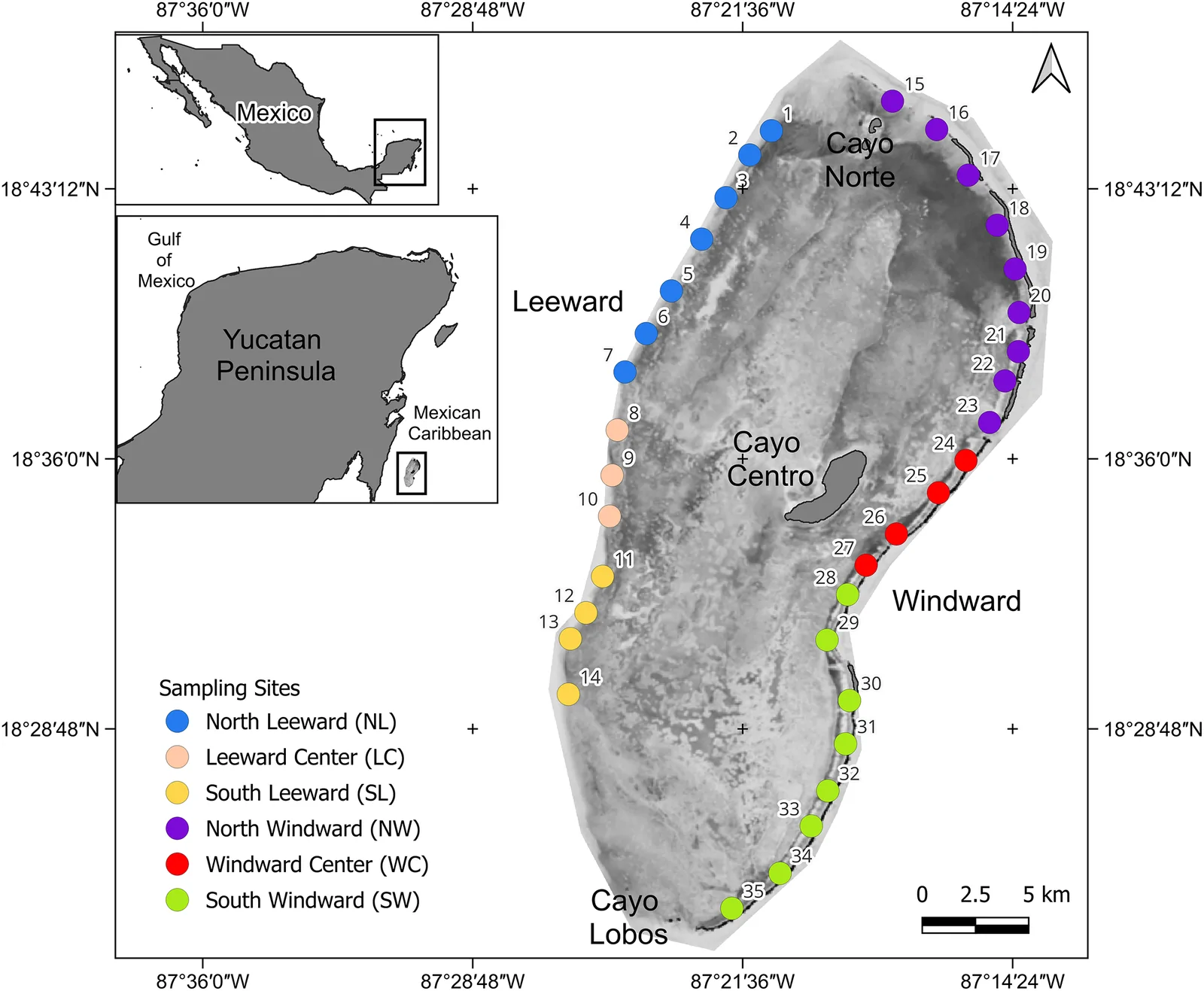
Location of the study area and distribution of the six zones considered in this study within the Banco Chinchorro Biosphere Reserve.

### Data collection

The survey was divided into three spatial levels. At the most coarse level surveys were divided into six Zones of greatest fishing importance for this species within the study area: North Leeward, Leeward Center, South Leeward, North Windward, Windward Center and South Windward (Fig. 1). Each Zone contained between three and nine stations, depending on its size, that were evenly distributed across the reef edge. At each station, five circular surveys were spaced 100 m apart, moving from the reef edge towards the center of the lagoon; there was not spatial heterogeneity between surveys within a station, which justifies pooling and analysis at a station level. Circular surveys were the finest spatial scale used in the survey and quantified the population size and structure within an 8 m radius. Divers collected queen conch specimens and transported them to the boat for counting and measurement. After measurements were completed, all individuals were carefully returned to the exact location where they were collected. Shell siphonal length (SL) and shell lip thickness (LT) were measured with a caliper to the nearest millimeter. SL was defined as the distance from the spire tip to the anterior edge of the shell. LT was defined as the distance from the mid-lateral region on the lip-side of the shell, approximately 40 mm from the edge of the shell (Carcamo, 2006). In 2015, surveys were carried out from August to November; in 2016, they were conducted in August, October, and November; and in 2018, they were performed in September and December. This working group held authorization from the National Commission of Aquaculture and Fisheries (CONAPESCA; Official Letter No. SDP/QR-23/319/2016) and from the National Commission of Natural Protected Areas (CONANP; Official Letter No. F00.9/DRBBCH/334/18) to carry out research activities without collecting specimens of species not considered at risk within the Banco Chinchorro Biosphere Reserve.

### Data analysis

#### Sampling design

The analysis was based on information obtained from sampling campaigns conducted in 2015, 2016, and 2018 (Table 1). The sampling design remained consistent throughout the study, with the same number of stations and replicates per station in each campaign. Differences in sampling effort were associated with the number of campaigns conducted per year and were taken into account during data processing and analysis to avoid bias in the interpretation of results. A total of 35 sampling stations were distributed across six zones of the reef lagoon. At each station, five circular surveys with an 8 m radius were conducted during each sampling campaign. Since individuals were not tagged or otherwise marked, some queen conch may have been recorded more than once across sampling events. Consequently, density estimates presented here do not represent the number of unique individuals in the population, but rather standardized measures of local abundance obtained from repeated surveys. These estimates were used to compare population density among zones and years and to assess broad-scale temporal changes in population status.

**Table 1:** Sampling design, effort, and sampling density by year.

Year	Field surveys (months)	Stations per survey	Replicates per station	Total replicates per year	Sampled area
2015	4	35	5	700	14.07
2016	3	35	5	525	10.55
2018	2	35	5	350	7.04
**Total**	9	–	–	1,575	31.66

#### Comparison of average density and siphonal length by zones and years

To compare mean density and size (SL) between zones and years, a one-way analysis of variance was performed (ANOVA; Statgraphics software). For the density analysis by zones, we first calculated the mean of the five replicates conducted at each station, taking into account each sampling month within each year. For the size analysis by zones, individuals were classified as suitable or not suitable for fishing using the minimum legal catch size in Mexico (200 mm SL) as the criterion: queen conch suitable for fishing (SL ≥ 200 mm) and queen conch not suitable for fishing (SL < 200 mm). Both density and SL data were log10-transformed prior to analysis to satisfy the assumptions of normality and homogeneity of variances required for ANOVA (Zar, 1999). When the null hypothesis of ANOVA was rejected, significant differences between zones and years were detected by Tukey’s test (HSD) for *post-hoc* comparisons (Zar, 1999).

Shell length (SL) data for queen conch classified as suitable and not suitable for fishing were arranged in a frequency table by zones. Zones that did not exhibit significant differences in mean SL, in both cases, were grouped for graphical representation.

#### Distribution of individuals according to siphonal length and lip thickness

In order to evaluate whether individuals suitable for fishing corresponded to sexually mature organisms, SL and LT values obtained over the 3 years were analyzed using a scatter plot between both variables. To determine the percentage of individuals in juvenile and adult phases, lip thickness (LT) was considered an indicator of sexual maturity: juveniles (LT < 15 mm) and adults (LT ≥ 15 mm) (Boman et al., 2018). However, regional-scale studies have shown that shell morphology including lip development varies spatially across the Caribbean, indicating that LT thresholds associated with maturity are not universal and should be calibrated at the local level. In particular, Boman et al. (2018) report marked differences among populations and morphotypes in the Caribbean. Since no prior histological information exists for the study area to establish an LT value associated with gonadal maturity, a threshold of 15 mm was used as an operational reference, acknowledging that this value represents an approximation and should be refined through future histological assessments.

## Results

### Comparison of average density and siphonal length by zones and years

Significant differences were found in mean density between zones in BCBR (ANOVA: F (5,174) = 29.34, *p* = 0.001). Tukey’s *post hoc* analysis grouped the zones into two groups and one individual zone: Leeward Center and South Leeward had the highest densities (1,095 ± 675 and 751 ± 674 ind. ha^−1^, respectively); Windward Center, North Windward and North Leeward showed intermediate densities (415 ± 385, 657 ± 614 and 574 ± 444 ind. ha^−1^, respectively); while South Windward recorded the lowest density (372 ± 355 ind. ha^−1^) (Fig. 2). Also, significant differences were observed between sampling years (ANOVA: F (2,224) = 9.83, *p* = 0.0001); the highest density was observed in 2016 (740 ± 684 ind. ha^−1^), it was followed by 2015 (600 ± 498 ind. ha^−1^) and the lowest density was observed in 2018 (360 ± 371 ind. ha^−1^) (Fig. 3).

**Figure 2:**
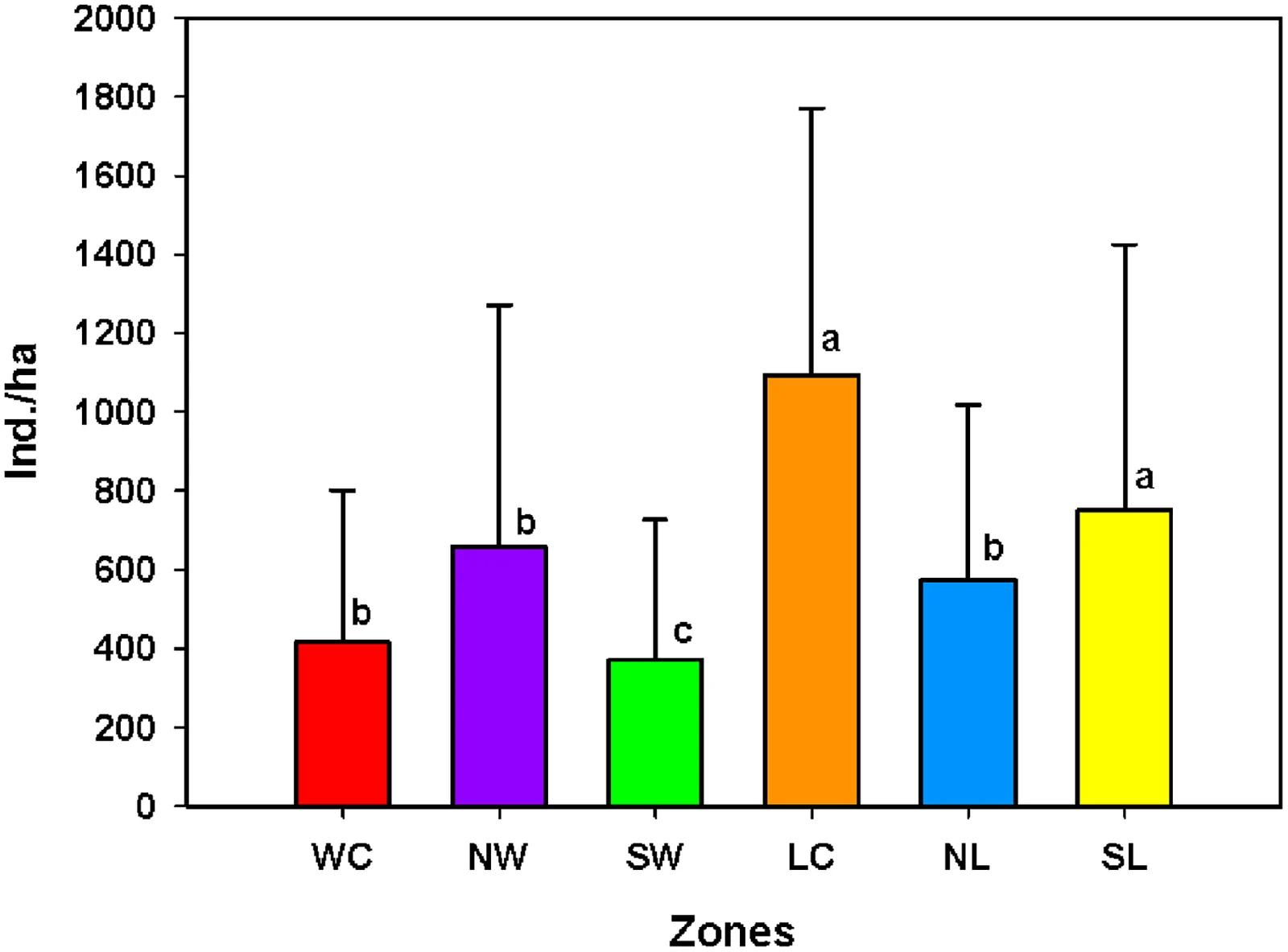
Comparison of the average density (±SD) of *Aliger gigas* (queen conch) across zones. Homogeneous groups (Tukey HSD test, *p* > 0.05) are denoted by identical letters for each treatment (zone): North Leeward (NL), Leeward Center (LC), South Leeward (SL), North Windward (NW), Windward Center (WC), and South Windward (SW).

**Figure 3:**
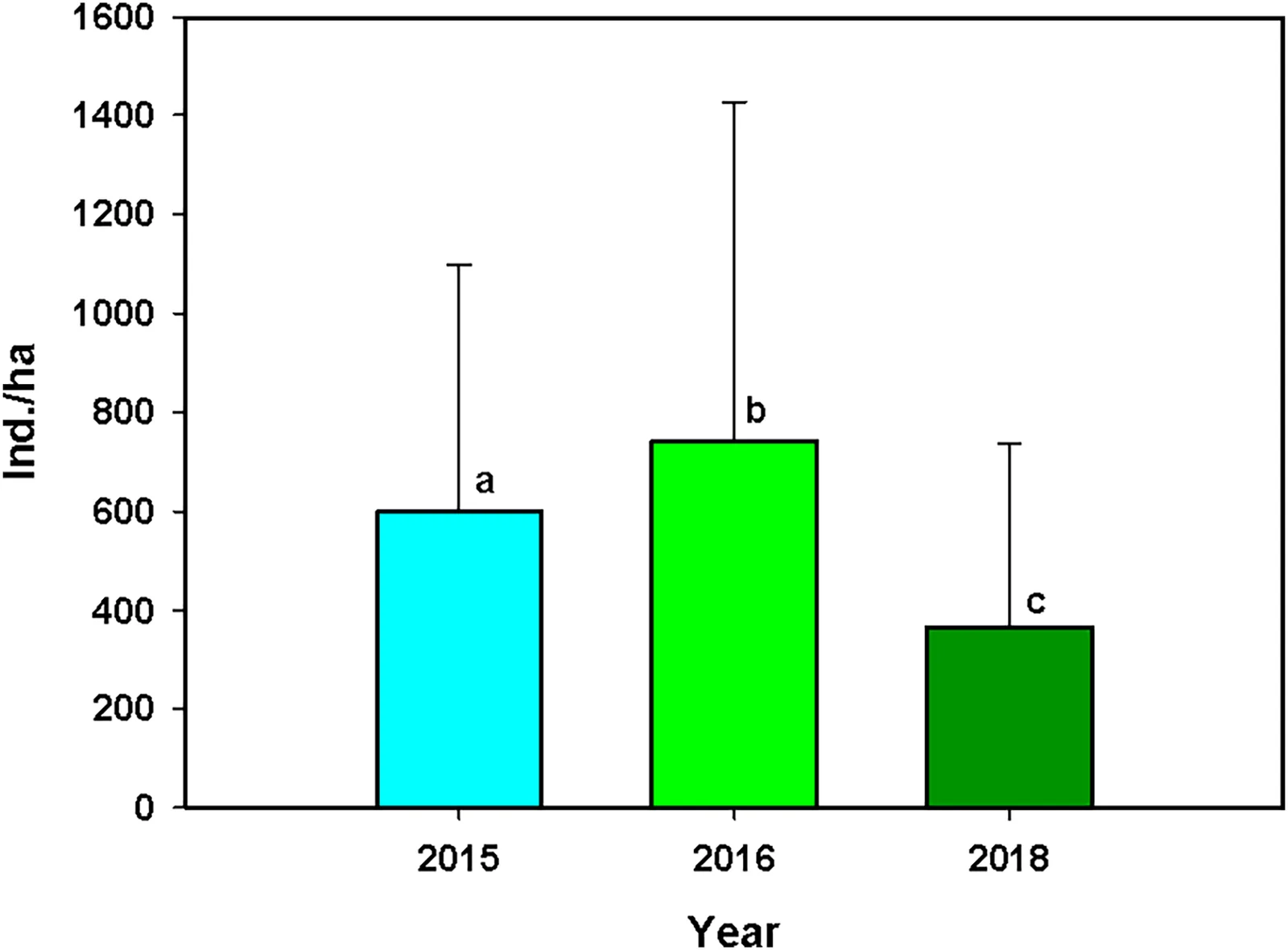
Comparison of the average density (± SD) of *Aliger gigas* (queen conch) by year. Homogeneous groups (Tukey HSD test, *p* > 0.05) are denoted by identical letters for each treatment (year).

Analysis of size distribution among zones, considering only individuals classified as not suitable for fishing (SL < 200 mm), revealed significant spatial differences across the BCBR (ANOVA: F (5, 13,454) = 91.14, *p* < 0.0001). Tukey’s *post hoc* test identified two distinct groups. The first group, comprising South Windward and Leeward Center, exhibited the smallest mean SL values (134.65 ± 15.25 mm and 137.25 ± 11.23 mm, respectively). In contrast, Windward Center, North Windward, North Leeward and South Leeward formed a second group characterized by intermediate SL values (157.23 ± 13.25 mm, 156.85 ± 13.52 mm, 156.99 ± 12.45 mm, and 154.64 ± 12.01 mm, respectively) (Fig. 4A).

**Figure 4:**
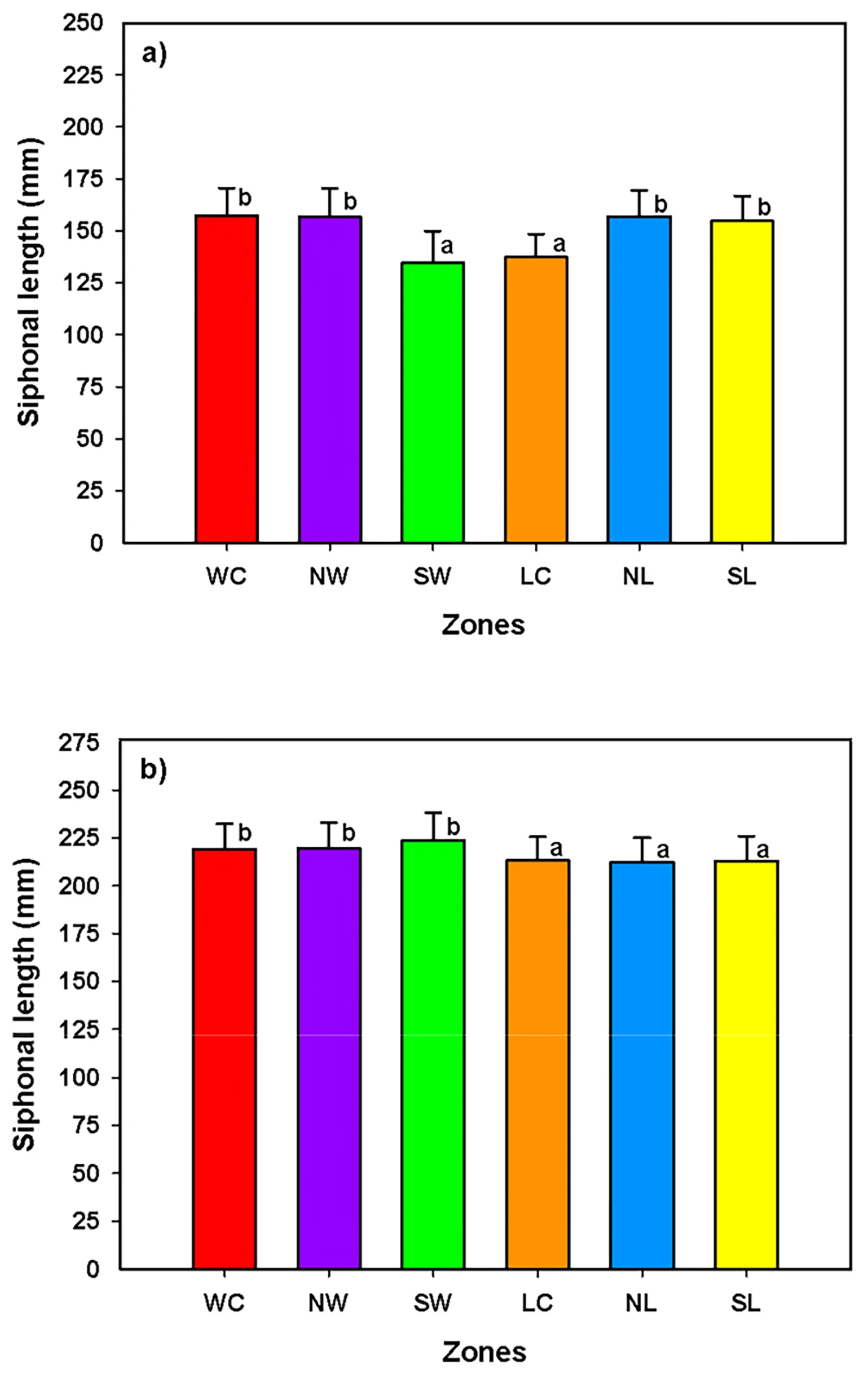
Comparison of the average siphonal length (SL) of *Aliger gigas* (queen conch) across zones: (A) individuals with SL < 200 mm; (B) individuals with SL ≥ 200 mm. Homogeneous groups (Tukey HSD test, *p* > 0.05) are denoted by identical letters for each treatment (zone): North Leeward (NL), Leeward Center (LC), South Leeward (SL), North Windward (NW), Windward Center (WC), and South Windward (SW).

When the analysis was restricted to individuals classified as suitable for fishing (SL ≥ 200 mm), significant differences among zones were also detected (ANOVA: F (5, 3,469) = 56.58, *p* < 0.0001). *Post hoc* comparisons distinguished two groups: a Windward group (Center, North, and South) characterized by the largest mean SL values (219.15 ± 13.22 mm, 219.34 ± 13.65 mm, and 223.55 ± 14.55 mm, respectively), and a Leeward group (Center, North, and South) with significantly smaller mean SL values (213.12 ± 12.45 mm, 212.21 ± 12.78 mm, and 212.87 ± 12.95 mm, respectively) (Fig. 4B). The lower mean values observed in the Leeward sector suggest populations dominated by specimens that have only recently attained harvestable size, whereas the Windward sector supported a greater proportion of larger individuals.

The SL frequency distribution of individuals below the legal catch size (SL < 200 mm) exhibited a bimodal pattern in both spatial groups, characterized by two well-defined modal classes. In group 1 (South Windward and Leeward Center; *n* = 4,537), the first mode was concentrated within the smaller size classes, whereas the second occurred in size classes approaching the minimum legal catch size (Fig. 5A). A similar pattern was observed in group 2 (Windward Center, North Windward, North Leeward and South Leeward; *n* = 8,917), where the distribution also displayed two frequency peaks separated by a relative decline in intermediate size classes. However, this group contained approximately three times more individuals than Group 1 (Fig. 5B).

**Figure 5:**
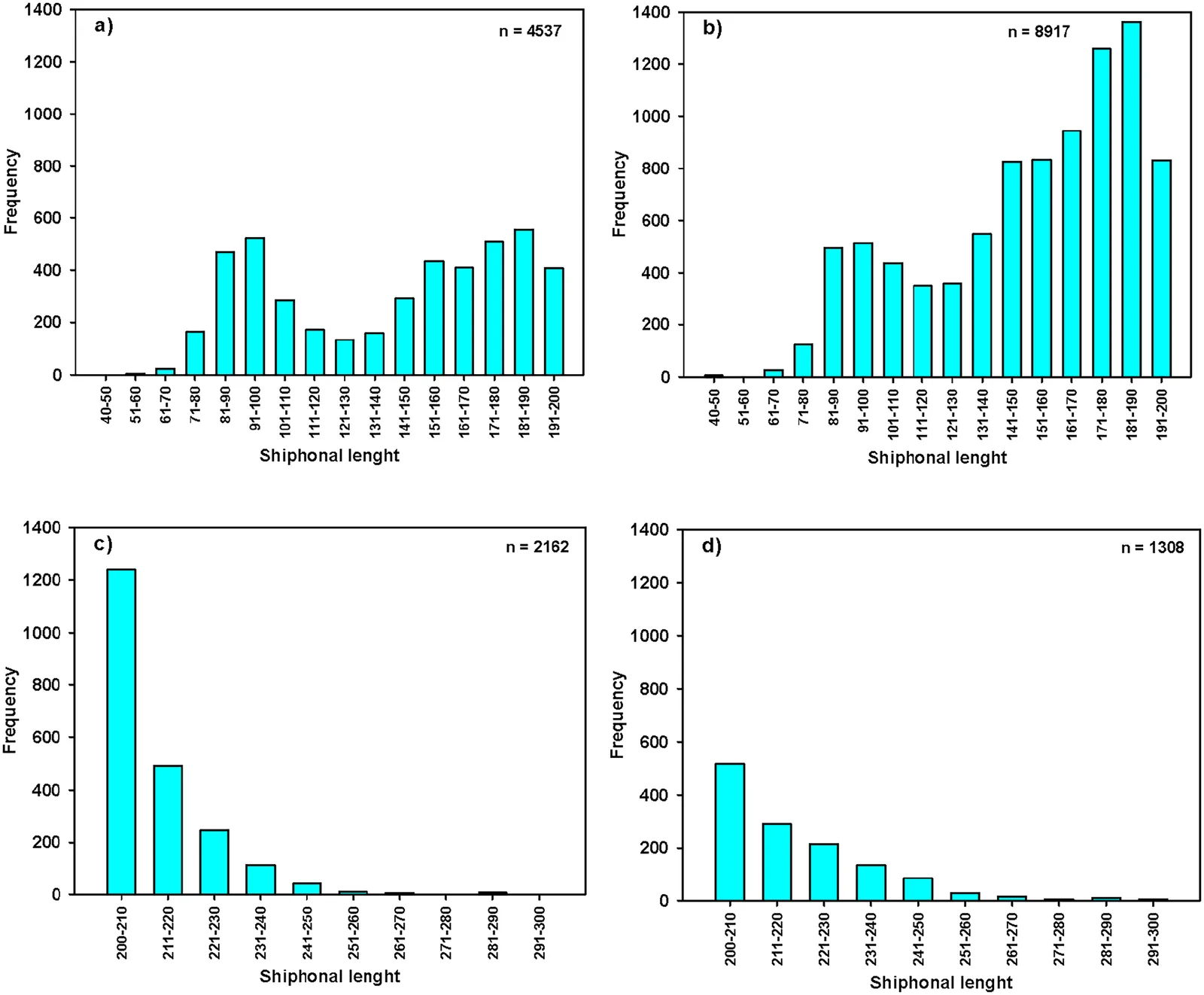
Size-frequency distribution (SL) of *Aliger gigas* (queen conch). Zones without significant differences were grouped according to siphonal length (SL). For individuals with SL < 200 mm as (A) group 1 (SW and LC) and (B) group 2 (WC, NW, NL, and SL); and for individuals with SL ≥ 200 mm as (C) group 1 (LC, NL, and SL) and (D) group 2 (WC, NW, and SW). North Leeward (NL), Leeward Center (LC), South Leeward (SL), North Windward (NW), Windward Center (WC), and South Windward (SW).

The size-frequency distribution of individuals above the legal catch size (SL ≥ 200 mm) was characterized by a predominance of organisms within size classes immediately above the minimum legal harvest threshold, followed by a progressive decline in frequency toward larger size classes. Group 1 (Leeward Center, North Leeward, and South Leeward; *n* = 2,162) exhibited the highest abundance of exploitable individuals and a broadly unimodal distribution, with most specimens concentrated in intermediate size classes and a low representation of large adults (Fig. 5C). A similar pattern was observed in group 2 (Windward Center, North Windward, and South Windward; *n* = 1,308), although the total abundance was substantially lower (Fig. 5D). In both groups, frequencies decreased markedly with increasing siphonal length, resulting in a limited proportion of large-sized individuals.

### Distribution of individuals according to siphonal length and lip thickness

Figure 6 shows the dispersion of the data from the three sampling years regarding the relationship between SL and LT; it is noticed that quadrant I hosts 1,089 (6.20%) organisms which are both suitable for fishing (SL ≥ 200 mm) and sexually mature (LT ≥ 15 mm); for its part, quadrant II contains a fairly large number of organisms (1,765; 10.03%) suitable for fishing (SL ≥ 200 mm), but which have not reached sexual maturity (LT < 15 mm).

**Figure 6:**
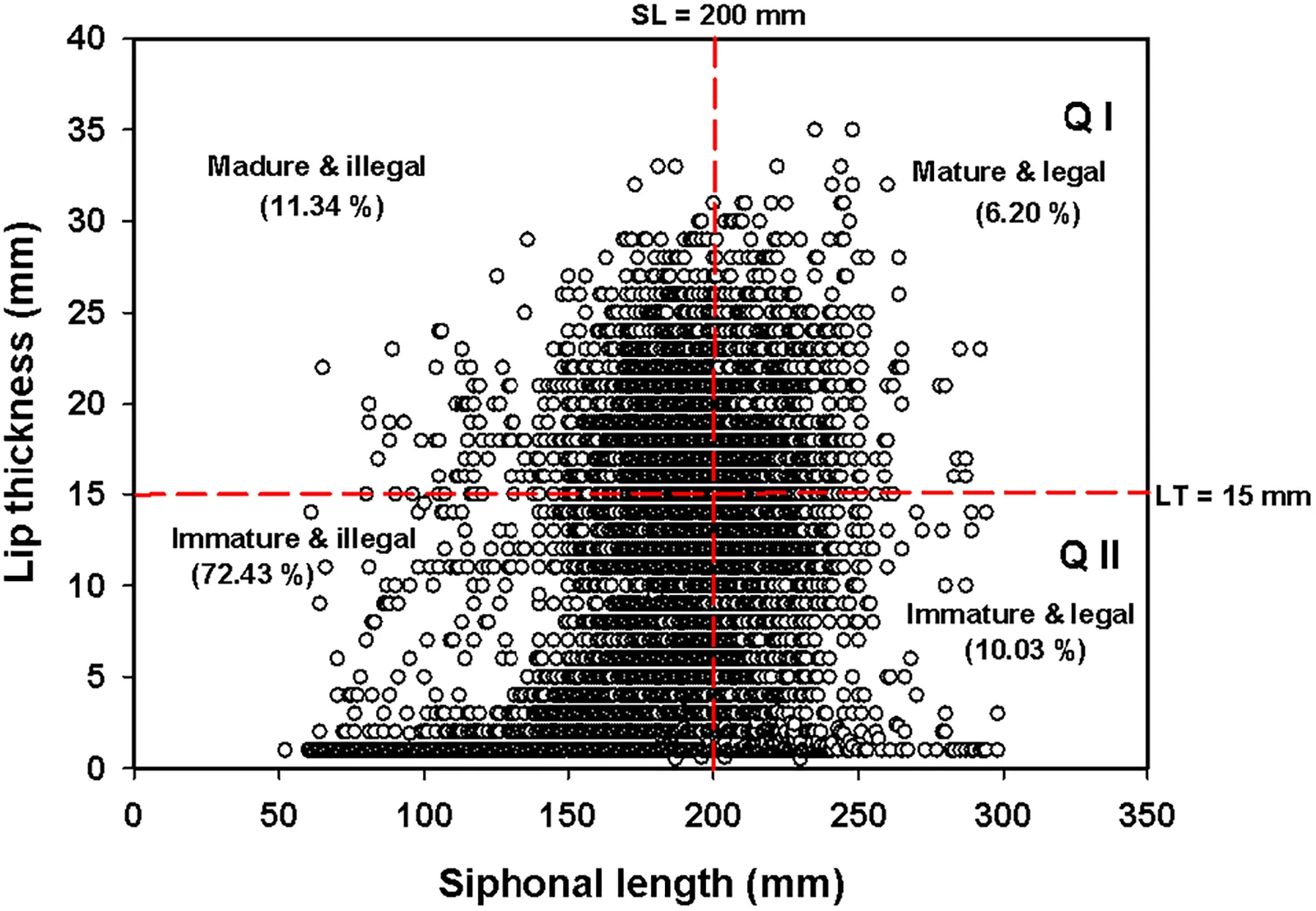
Scatter plot showing the relationship between siphonal length and lip thickness in the *Aliger gigas* (queen conch) population from Banco Chinchorro, Mexico. Quadrant I (Q I) group individuals of legal size and biological maturity (LT ≥ 15 mm), whereas Quadrant II (Q II) comprises individuals of legal size (SL ≥ 200 mm) that are still immature.

## Discussion

This study provides one of the most comprehensive assessments of the population status of *A. gigas* within the BCBR after the implementation of a 5-year fishery closure. Overall, the results indicate a partial recovery in population density relative to previous estimates, suggesting a positive response to the temporary cessation of harvesting. However, this recovery was not accompanied by a proportional increase in the abundance of sexually mature adults, as the population remained strongly dominated by juvenile and pre-adult individuals. Furthermore, a substantial proportion of individuals exceeding the legal minimum harvest size had not yet attained sexual maturity based on lip thickness criteria, highlighting a potential mismatch between current management regulations and the biological characteristics of the population. Together, these findings suggest that although the closure may have contributed to increasing abundance, important challenges remain for rebuilding the reproductive stock and ensuring the long-term sustainability of the fishery.

### Comparison of average density and siphonal length by zones and years

In this study, the highest densities of queen conch were recorded in the Leeward Center and South zones. Previous studies (Domínguez-Viveros, Sosa-Cordero & Medina-Quej, 1994; De Jesús-Navarrete, Medina-Quej & Oliva-Rivera, 2003) reported high densities of juveniles in the northern and central zones, while the Lobos and Isla Che zones (in the south of the study area) exhibited low densities and predominance of adult individuals. However, subsequent research work (De Jesús-Navarrete & Valencia-Hernández, 2013; Cala et al., 2013) documented an inverse pattern, with higher densities of juvenile and adult individuals in the south of BCBR, attributed to a greater depth, which hinders free-dive catch. In this study, the high density observed in the Leeward Center zone may reflect a population recovery after the 5-year closure, particularly in this juvenile recruitment zone where fishing pressure is minimal. The spatial distribution of queen conch density in the BCBR has shown significant changes in recent decades. These changes might be associated with the historical fishing effort concentrated in the northern portion of the reef, characterized by shallow areas with seagrasses, which facilitated the intensive extraction of the resource. In contrast, the greater depth and structural complexity of the southern portion made its capture difficult, which favored its conservation. This pattern is consistent with long-term observations from The Bahamas, where adult queen conch densities and age structure were strongly influenced by fishing pressure, while deeper and less accessible habitats retained older and more abundant populations (Stoner, Davis & Kough, 2018).

The spatial distribution of queen conch may also be influenced by ontogenetic and environmental factors. Along their development, young specimens migrate from shallow habitats with sandy substrates and microalgae to deeper areas with seagrasses and macroalgae, eventually reaching channels and areas of deeper reef structures as they become sexually mature (Stoner, 2003; Doerr & Hill, 2018; Tewfik et al., 2019). Additionally, climate-related stressors, including increasing sea surface temperatures, ocean acidification, and the widespread influxes of *Sargassum* across the Caribbean, may affect the distribution and survival of queen conch populations by altering water quality and degrading seagrass and macroalgal habitats (Oxenford & Monnereau, 2017; Hernández-Delgado, 2023; Martinez-Ortiz et al., 2025). However, the response of queen conch populations to habitat degradation appears to be context dependent. Telemetry studies conducted in Xel-Há, Mexico, demonstrated that *A. gigas* can persist in marginal coastal habitats characterized by recurrent low-oxygen conditions, exhibiting movement patterns and habitat use comparable to those observed in more typical marine environments (Stieglitz & Dujon, 2017). Furthermore, these habitats may function as important nursery areas that contribute to population persistence and connectivity through ontogenetic migrations toward offshore habitats (Stieglitz et al., 2020). Therefore, the effects of environmental degradation on queen conch populations are likely mediated by local habitat conditions, connectivity, and population dynamics.

The temporal analysis of the population density of queen conch density showed significant differences between sampling years. Three years after the 5-year ban came into force, a recovery trend was observed in 2015, which intensified in 2016. However, in 2018 the density decreased after the authorization of a 9-tonne catch quota for the three fishing cooperatives that operate in the BCBR. This recovery during the ban was reported by Sabido-Itzá et al. (2021), who report an increase in CPUE from 5.09 to 8.0 kg of meat/fisherman/hour between the 2011 (pre-ban) and 2018 (post-ban) fishing seasons, suggesting a greater abundance of the resource after 5 years of protection.

The spatial variation observed in SL among individuals classified as not suitable for fishing (SL < 200 mm) reveals marked heterogeneity in the population structure of *A. gigas* throughout the BCBR. The differences detected among zones suggest that recruitment, growth, and early survival processes do not occur uniformly across the reserve. In particular, South Windward and Leeward Center exhibited the smallest mean sizes, indicating a greater representation of individuals in early juvenile stages. This pattern is consistent with the spatial distribution previously described for the species in Banco Chinchorro, where the Leeward Center zone has historically been identified as an important juvenile recruitment area (Domínguez-Viveros, Sosa-Cordero & Medina-Quej, 1994; Cala et al., 2013). The concentration of small individuals in these sectors suggests that they continue to function as essential settlement and nursery habitats, contributing recruits to the broader population within the reserve. The occurrence of smaller individuals in South Windward is particularly noteworthy, as this area had not previously been recognized as a major recruitment habitat. Nevertheless, the habitat characteristics observed in this zone, including relatively shallow depths, sandy substrates, and extensive coverage of macroalgae and seagrass beds, are consistent with conditions considered optimal for the settlement and development of juvenile *A. gigas*. Studies conducted throughout the Caribbean have shown that early juveniles exhibit a strong association with seagrass meadows and sheltered sandy bottoms, habitats that provide both refuge from predators and adequate food resources for growth (Stoner et al., 1994; Stoner, 2003). Consequently, the size structure documented in this study suggests that South Windward may represent a previously undescribed nursery area within the reserve.

In contrast, Windward Center, North Windward, North Leeward, and South Leeward were characterized by significantly larger individuals, although they still fell within the category of individuals not suitable for fishing. The predominance of late juveniles and subadults in these localities suggests that these sectors may function as intermediate growth habitats, where individuals remain after the initial recruitment phase and prior to entering the adult population. The persistence of this pattern through time further indicates spatial stability in the ecological role of these areas as developmental habitats for advanced juvenile stages of the species.

The spatial differences observed among harvestable individuals (SL ≥ 200 mm) indicate that the demographic structure of *A. gigas* is likewise heterogeneous across the BCBR. The larger mean SL values recorded in the Windward zones suggest a greater representation of older and potentially more mature individuals, whereas the smaller sizes observed in the Leeward sector indicate populations composed primarily of organisms that have only recently attained the legal harvest size. Similar patterns have been documented in other Caribbean populations and have been associated with ontogenetic movements and habitat specific environmental conditions (Appeldoorn, 1994; Stoner et al., 1994; Stoner, Davis & Booker, 2012). This Windward-Leeward gradient may be linked to both ontogenetic shifts and environmental variability. The Windward sector is exposed to greater hydrodynamic influence resulting from prevailing trade winds and regional ocean circulation (De Jesús-Navarrete, 2003; Comisión Nacional de Áreas Naturales Protegidas (CONANP), 2006), factors that can affect food availability, sediment dynamics, and habitat structure. Given that the growth and distribution of *A. gigas* are closely associated with local habitat characteristics, including substrate type, seagrass cover, algal biomass, and hydrodynamic regimes (Stoner & Waite, 1991; Glazer, Delgado & Kidney, 2003; Prada et al., 2017), these conditions may favor both the growth and persistence of larger individuals within Windward habitats. Alternatively, these habitats may become increasingly attractive to individuals as they mature and undergo ontogenetic habitat shifts. Previous studies have documented spatial movements and habitat-specific use patterns throughout the life cycle of *A. gigas* (Stoner, 2003; Glazer, Delgado & Kidney, 2003; Stieglitz & Dujon, 2017; Stieglitz et al., 2020). However, the environmental cues underlying these movements remain poorly understood. Therefore, the possibility that hydrodynamic conditions contribute to habitat selection is presented here as a hypothesis requiring further investigation rather than as a demonstrated mechanism. From a reproductive perspective, this pattern is particularly relevant because fecundity in *A. gigas* increases with adult body size (Appeldoorn, 2020).

The temporal analysis of the mean SL of the queen conch, considering the three sampling years, did not reveal a consistent pattern that could be directly associated with the implementation of the fishery closure. The interannual variations observed in this variable are primarily explained by changes in the population’s age structure particularly the relative proportion of juveniles, pre-adults, and adults rather than by biologically or demographically meaningful population-level shifts.

The bimodal size-frequency distribution observed in individuals below the legal catch size 200 mm (SL < 200 mm) in both groups suggests the presence of at least two distinct juvenile cohorts, likely associated with recruitment pulses occurring at different times. This pattern is consistent with the population dynamics described for *A. gigas*, in which variability in recruitment, together with differences in growth and survival during early life stages, results in distinguishable cohorts within size-frequency distributions (Peel & Aldana-Aranda, 2012; Stoner, Davis & Booker, 2012). The higher abundance of juveniles recorded in Group 2 may indicate more favorable conditions for larval settlement and early development, highlighting the importance of these areas as potential nursery habitats (Stoner, 2003).

In contrast, the size structure of individuals above the legal catch size (SL ≥ 200 mm) was characterized by a high concentration of individuals in size classes immediately above the minimum legal harvest size and a progressive decline toward larger size classes. This pattern is typical of populations subjected to fishing pressure, where the selective removal of larger individuals truncates the population size structure and reduces the proportion of large adults within the stock (Stoner, Davis & Booker, 2012; Stoner & Appeldoorn, 2021). The low frequency of large individuals observed in both groups is particularly relevant because larger adults contribute disproportionately to the reproductive output of the population. In *A. gigas*, fecundity increases with both body size and age; therefore, the reduction of these size classes may decrease egg production and limit the stock’s recovery potential (Stoner & Appeldoorn, 2021). Furthermore, the decline in reproductive adults may intensify density-dependent processes, reducing mating success and overall reproductive performance in exploited populations (Stoner & Ray-Culp, 2000).

Overall, the results indicate a population characterized by active recruitment and a substantial representation of juveniles, but a limited proportion of large individuals within the exploitable fraction. This combination suggests that, although the incorporation of new individuals into the population appears to be ongoing, the size structure of adults may be affected by selective fishing. Consequently, beyond enforcing the minimum legal harvest size, management efforts should prioritize the conservation of reproductive adults due to their critical role in maintaining population reproductive capacity (Stoner & Appeldoorn, 2021). Likewise, the protection of essential habitats that support recruitment and juvenile development is fundamental to ensuring the long-term sustainability of *A. gigas* populations (Prada et al., 2017).

### Distribution of individuals according to siphonal length and lip thickness

The scatter plot showed that only 6.20% of the sampled individuals (*n* = 1,089) were classified as above the legal catch size (SL ≥ 200 mm) and simultaneously exhibited the LT threshold considered indicative of sexual maturity (LT ≥ 15 mm). In contrast, 10.03% (*n* = 1,765) of the sampled individuals were above the legal catch size (SL ≥ 200 mm) but did not meet the operational maturity criterion (LT < 15 mm). These findings suggest that a substantial proportion of the *A. gigas* population in the BCBR may be vulnerable to harvest before contributing to reproduction. This observation is consistent with previous studies indicating that shell size, as measured by siphonal length (SL), does not necessarily reflect the reproductive status of individuals, because longitudinal shell growth may be completed before gonadal maturation occurs. In contrast, lip development and thickening have been identified as morphological traits more closely associated with age and the transition to adulthood and reproductive maturity (Stoner et al., 2012; Foley & Takahashi, 2017; Tewfik et al., 2019). Furthermore, the relationship between shell size and maturity has been shown to vary among populations as a result of environmental and geographic influences, suggesting that management measures based solely on shell length may not adequately protect reproductive individuals (Foley & Takahashi, 2017; Boman et al., 2018).

However, the LT ≥ 15 mm threshold used in this study should be regarded as an operational reference rather than a definitive maturity criterion for the local population. Boman et al. (2018) demonstrated that LT associated with sexual maturity can vary among *A. gigas* populations and morphotypes throughout the Caribbean, indicating that thresholds derived from one region may not be directly applicable to another. Consequently, although the present results reveal a discrepancy between individuals above the legal catch size (SL ≥ 200 mm) and their potential maturity status, validation of these patterns requires local histological studies to establish biologically meaningful maturity thresholds. In this regard, Stoner & Appeldoorn (2021) recommended the estimation of parameters such as LT_min_ and LT_50_ to relate LT to gonadal maturity and to develop biologically informed management criteria. Therefore, the findings of this study highlight the need to complement minimum harvest-size regulations with biological indicators of maturity that ensure more effective protection of the reproductive potential of *A. gigas*.

## Conclusions

This study provides robust evidence that the 5-year fishery closure implemented in the BCBR contributed to a partial recovery of *A. gigas* population density, reaching levels higher than those reported during the period of greatest population decline. Nevertheless, the demographic structure remained strongly skewed toward juveniles and subadults, while sexually mature adults represented only a small fraction of the population, indicating that recovery in abundance has not yet translated into a full restoration of reproductive biomass. Spatial patterns in density and size structure revealed the existence of functionally distinct habitats within the reserve, including areas that appear to support recruitment, juvenile growth, and the persistence of larger individuals. These findings underscore the importance of maintaining habitat heterogeneity and protecting key nursery and adult habitats to sustain population connectivity and replenishment processes. A particularly important result is the discrepancy between legal harvest size and biological maturity. More than 10% of individuals exceeded the minimum legal SL but had not reached the LT threshold associated with sexual maturity, whereas only 6.2% fulfilled both criteria simultaneously. This finding suggests that regulations based exclusively on shell length may allow the removal of individuals before they contribute effectively to reproduction, thereby reducing the reproductive potential of the stock.

Overall, our results indicate that population recovery in *A. gigas* should be evaluated not only in terms of abundance, but also through demographic and reproductive indicators. Incorporating biological maturity criteria, such as LT, into management regulations, together with the protection of critical habitats and continued population monitoring, would strengthen conservation strategies and enhance the long-term sustainability of queen conch fisheries in the BCBR and throughout the Caribbean. Future studies should integrate histological assessments of maturity and long-term demographic monitoring to refine harvest regulations and improve the conservation of this ecologically and economically important species.

Finally, density estimates were analyzed using station-level averages to minimize pseudoreplication among adjacent surveys and focus on broad-scale spatial patterns. Although this approach may underestimate fine-scale variability, it provides a robust basis for reserve-scale management. Future studies using hierarchical or mixed-effects models could improve the resolution of population assessments.

## Supplemental Information

10.7717/peerj.21836/supp-1 Supplemental Information 1 Biological database.

## Additional Information and Declarations

### Field Study Permissions

The following information was supplied relating to field study approvals (*i.e*., approving body and any reference numbers):

Comisión Nacional de Areas Naturales Protegidas (F00.9/DRBBCH/334/18).

### Data Availability

The following information was supplied regarding data availability:

The raw data is available in the Supplemental Files.
